# Unraveling 14-3-3 Proteins in C_4_ Panicoids with Emphasis on Model Plant *Setaria italica* Reveals Phosphorylation-Dependent Subcellular Localization of RS Splicing Factor

**DOI:** 10.1371/journal.pone.0123236

**Published:** 2015-04-07

**Authors:** Karunesh Kumar, Mehanathan Muthamilarasan, Venkata Suresh Bonthala, Riti Roy, Manoj Prasad

**Affiliations:** National Institute of Plant Genome Research (NIPGR), New Delhi, India; RIKEN Center for Sustainable Resource Science, JAPAN

## Abstract

14-3-3 proteins are a large multigenic family of regulatory proteins ubiquitously found in eukaryotes. In plants, 14-3-3 proteins are reported to play significant role in both development and response to stress stimuli. Therefore, considering their importance, genome-wide analyses have been performed in many plants including *Arabidopsis*, rice and soybean. But, till date, no comprehensive investigation has been conducted in any C_4_ panicoid crops. In view of this, the present study was performed to identify 8, 5 and 26 potential *14-3-3* gene family members in foxtail millet (*Si14-3-3*), sorghum (*Sb14-3-3*) and maize (*Zm14-3-3*), respectively. *In silico* characterization revealed large variations in their gene structures; segmental and tandem duplications have played a major role in expansion of these genes in foxtail millet and maize. Gene ontology annotation showed the participation of 14-3-3 proteins in diverse biological processes and molecular functions, and *in silico* expression profiling indicated their higher expression in all the investigated tissues. Comparative mapping was performed to derive the orthologous relationships between *14-3-3* genes of foxtail millet and other Poaceae members, which showed a higher, as well as similar percentage of orthology among these crops. Expression profiling of *Si14-3-3 *genes during different time-points of abiotic stress and hormonal treatments showed a differential expression pattern of these genes, and sub-cellular localization studies revealed the site of action of Si14-3-3 proteins within the cells. Further downstream characterization indicated the interaction of Si14-3-3 with a nucleocytoplasmic shuttling phosphoprotein (SiRSZ21A) in a phosphorylation-dependent manner, and this demonstrates that Si14-3-3 might regulate the splicing events by binding with phosphorylated SiRSZ21A. Taken together, the present study is a comprehensive analysis of *14-3-3* gene family members in foxtail millet, sorghum and maize, which provides interesting information on their gene structure, protein domains, phylogenetic and evolutionary relationships, and expression patterns during abiotic stresses and hormonal treatments, which could be useful in choosing candidate members for further functional characterization. In addition, demonstration of interaction between Si14-3-3 and SiRSZ21A provides novel clues on the involvement of 14-3-3 proteins in the splicing events.

## Introduction

14-3-3 proteins are large multigenic family of regulatory proteins which are ubiquitously present in all eukaryotes. They regulate plant development and defense from stresses through protein-protein interactions by binding with phosphoserine / phosphothreonine residues in target proteins [1–3]. These 14-3-3 proteins interact as a dimer with native dimeric size of ~60 kDa where each monomer in dimer can interact with separate target proteins [3]. This facilitates 14-3-3 dimer to act as a scaffolding protein to structurally modify the target protein. Noteworthy, hundreds of targets for 14-3-3 proteins have been identified in plants which are involved in different cellular processes such as gene expression, protein synthesis, hormone signaling and primary metabolism including plasma membrane located H^+^-ATPase [3,4]. Although animals have seven 14-3-3 proteins, plants possess a varying number of isoforms showing their evolutionary divergence [5]. *Arabidopsis* encodes 13 isoforms of 14-3-3 [6,7], whereas rice has 8 [8], 5 are reported in barley [9], 17 isoforms in tobacco [10], 6 in cotton [11] and 18 in soybean [3].

Though the role of plant 14-3-3 proteins in key physiological processes including metabolism (particularly, primary carbon and nitrogen metabolism), and development had been well reported, recent evidences have shown the participation of 14-3-3 proteins in abiotic and biotic stress response pathways [5]. These studies on the role of 14-3-3 in stress tolerance revealed their versatility, such as (i) *14-3-3* gene expression is modified by diverse stress stimuli [12], (ii) 14-3-3 proteins interact with the components of stress signaling pathways [13], (iii) transgenic plants with modified *14-3-3* expression displayed differential stress responses [14] and (iv) 14-3-3 proteins undergo self-phosphorylation by stress-activated kinases [15]. Keeping in view the importance of 14-3-3 proteins, genome-wide analysis of this multigene family has been conducted in C_3_ crops such as *Arabidopsis* [6], rice [8] and soybean [3], but no such studies have been performed in C_4_ panicoid crop species.

C_4_ crops are equipped with phosphoenolpyruvate carboxylase (PEPC) which performs immediate quenching and delivery of carbon dioxide to RuBisCO, thus resulting in faster photosynthesis, even under high light and elevated temperatures. In addition, this swift processing of PEPC does not require the opening of stomata for a longer period, ultimately leading to decreased transpiration levels. Taken together, C_4_ crops are efficient in photosynthesis and possess better water use efficiency (WUE) [16,17]. Hence, deciphering *14-3-3* gene families in sequenced C_4_ panicoid genomes namely foxtail millet, sorghum and maize would enable functional characterization of these genes for gaining knowledge on the role of 14-3-3 proteins in C_4_ crop physiology and stress response. Foxtail millet (*Setaria italica* L.) has been recognized as a model crop for investigating the genetics and genomics of C_4_ panicoid crops [16–19]. A comparative transcriptomic analysis of differentially expressed genes in dehydration tolerant and susceptible cultivars of foxtail millet revealed a relatively higher expression of *14-3-3* transcripts in tolerant cultivar [20], thus supporting the speculation that 14-3-3 proteins might play a crucial role in stress tolerance behaviour of foxtail millet. These 14-3-3 proteins are phosphopeptide binding proteins and their binding motif have been identified in serine/arginine (SR) domain containing splicing factor which actively participate in pre-mRNA splicing [21]. SR proteins are well conserved non-small nuclear ribonucleoprotein (non-snRNP) splicing factors, characterized by their modular organization and presence of a RNA recognition motif (RRM), a single CCHC-zinc knuckle motif and a RS domain towards C-terminus end of proteins [21]. Human SR proteins are localized in nucleus, whereas three of them SF2/ASF, SRp20, and 9G8 shuttle continuously between nucleus and cytoplasm due to RS domain phosphorylation [22]. Phosphorylation of RS domain may affect protein-protein interaction as well as localization of RS proteins [23,24]. Although these reports indicate that phosphorylation of RS domains is mainly involved in nucleocytoplasmic shuttling, no study has identified how phosphorylation affects the location of RS splicing factors. Considering this, the present study was performed to understand the role of 14-3-3 in dynamicity of RS splicing factor.

## Materials and Methods

### *In silico* identification and annotation of *14-3-3* family genes in sequenced C_4_ panicoids

Hidden Markov Model (HMM) profile of 14-3-3 domain (PF00244) downloaded from Pfam v27.0 (http://Pfam.sanger.ac.uk/) was used to identify 14-3-3 proteins in *Setaria italica* (Si14-3-3), *Sorghum bicolor* (Sb14-3-3) and *Zea mays* (Zm14-3-3) following Mishra et al. [25]. Subsequently, *14-3-3* encoding genes were identified through BLAST search against respective genomes available in Phytozome (http://www.phytozome.net/). HMMSCAN was performed to confirm the presence of conserved 14-3-3 domain in all identified 14-3-3 proteins (http://hmmer.janelia.org/search/hmmscan), and their physicochemical properties were identified using ExPASy ProtParam tool (http://web.expasy.org/protparam/). Physical mapping as well as identification of tandem and segmental duplications was performed using the methods described elsewhere [26]. Intron-exon organization of identified *14-3-3* genes was analyzed using Gene Structure Display Server v2.0 (http://gsds.cbi.pku.edu.cn) and phylogenetic tree was constructed using MEGA6 as described in Lata et al. [27]. MEME tool (http://meme.nbcr.net/meme3/meme.html) was used to predict conserved protein sequence domains following the parameters mentioned in Puranik et al. [28]. Gene Ontology annotation was performed using Blast2GO v2.7.1 [29] and promoter analysis was done through PLACE database (http://www.dna.affrc.go.jp/PLACE/).

Illumina RNA-HiSeq reads from 4 tissues of foxtail millet [spica (SRX128226), stem (SRX128225), leaf (SRX128224), and root (SRX128223)], 8 tissues of sorghum [leaf (SRR349644), emerging inflorescence (SRR349645), seed (SRR349646), early inflorescence (SRR349754), pistil (SRR349767), embryo (SRR349768), endosperm (SRR349769) and anther (SRR349769)], and 2 tissues of maize [leaf base (SRR029157) and leaf tip (SRR029158)] retrieved from European Nucleotide Archive (http://www.ebi.ac.uk/ena) were used to analyze tissue-specific expression of *14-3-3* genes following the procedure described in Yadav et al. [30]. Comparative mapping of foxtail millet *14-3-3* genes with orthologs in *S*. *bicolor*, *Z*. *mays*, *O*. *sativa* and *B*. *distachyon* genomes was performed following Muthamilarasan et al. [17] and estimation of synonymous and non-synonymous substitution rates was carried out following Puranik et al. [28].

### Plant materials, stress treatments and expression profiling

Abiotic stress tolerant foxtail millet cultivar ‘Prasad’ was used in the present study. The seeds were obtained from National Bureau of Plant Genetic Resources (NBPGR), Hyderabad, India and grown in plant growth chamber following the conditions mentioned by Puranik et al. [28]. Twenty one-day-old seedlings were exposed to stress [250 mM NaCl (salinity), 20% PEG 6000 (dehydration)] and hormonal treatments [100 μM abscisic acid (ABA), 100 μM salicylic acid (SA), 100 μM methyl jasmonate (MJ)] following Muthamilarasan et al. [17]. The whole seedlings were sampled at 1, 3, 12, 24 and 48 hours (hr) [25,28]. The samples were immediately frozen in liquid nitrogen and stored at -80°C until RNA isolation. Unstressed plants were maintained as controls and the experiment was performed in triplicate. RNA isolation, quantitation, cDNA synthesis, qRT-PCR analysis (three technical replicates for each biological duplicate) and determination of relative transcript levels were performed according to Muthamilarasan et al. [17] and Mishra et al. [25], using the primers mentioned in S1 Table.

### Subcellular localization of *Si14-3-3* genes

Vector *pAMPAT* was selected for YFP-tagged expression, and primers used for cloning are given in S1 Table. Amplifications were performed using Phusion High-fidelity DNA polymerase (Thermo Fisher Scientific) with the following program: denaturation at 94°C for 3 min; followed by 35 cycles of 94°C for 0.5 min, 60°C for 0.5 min, and 72°C for 1 min; final extension at 72°C for 10 min. PCR amplified fragment was gel purified for cloning into pGEM-T Easy Vector (Promega). After transformation in *Escherichia coli* strain DH5α, the inserts were verified by sequencing (ABI3730xl DNA Analyzer). The positive clones were digested with selected restriction enzymes and digested fragment was gel purified and ligated to *pAMPAT* pre-digested with the same enzymes to create a *pAMPAT-14-3-3* for translational fusion with C-terminus of YFP tag. The fusion plasmids were then introduced into onion peel using Biolistic PDS-1000/He Particle Delivery System (Bio-Rad) and fluorescence was visualized using laser confocal scanning microscope (Leica Microsystems). The laser and pinhole settings of confocal microscope were kept identical throughout the study.

### Yeast two-hybrid assay and bimolecular fluorescent complementation analysis

*Si14-3-3_f* and *SiRSZ21A* genes were cloned into pGADT7 and pGBKT7 vectors, respectively, using gene specific primers (S1 Table) and the chimeric vectors were co-transformed in Y2HGold Yeast Strain by PEG/LiAc method according to manufacturer’s instructions (Clontech). For BiFC analysis, full length CDS of *Si14-3-3_f* and *SiRSZ21A* were amplified using primers (S1 Table) and cloned into pENTR/D-TOPO vector (Invitrogen) according to manufacturer’s instructions. Si14-3-3_f-pENTR and SiRSZ21A-pENTR were then recombined with destination vector pBiFP2 (C-terminal half of YFP) and pBiFP3 (N-terminal half of YFP), respectively, using LR clonase enzyme mix (Invitrogen). Upon confirmation by sequence analysis, positive clones were introduced into onion epidermis and *Nicotiana benthamiana* leaves using PDS-1000/He system (BioRad) and *Agrobacterium*-mediated transformation, respectively.

### Site-directed mutagenesis (SDM)

To study phosphorylation-dependent interactions, *SiRSZ21A* was cloned into pENTR/D-TOPO vector and positive recombinant plasmids were quantified spectrophotometrically. For SDM, primers were designed in such a way that 3 nucleotides were changed at 118, 120 and 124 positions of SiRSZ21A protein corresponding to serine which has to be converted into alanine residues *m1* (S120→A120), *m2* (S122→A122) and *m3* (S124→A124). Both forward and reverse primers were designed complimentary to each other (S1 Table). PCR amplification was performed using a reaction mixture comprising 25 ng of plasmid DNA, 1.5 pmol of each primer, 2.5 mM dNTPs, 10X *Pfu* buffer containing 15mM MgCl_2_ and 2.5 U of *Pfu* polymerase. PCR profile was: 30 sec initial denaturation at 94°C, 18 cycles of 30 sec denaturation at 94°C, 60 sec annealing at 55°C followed by 16 min extension at 72°C. Amplified products were digested with restriction enzyme *Dpn* I at 37°C overnight and transformed into *E*. *coli* DH5α competent cells.

## Results

### 14-3-3 proteins of C_4_ panicoids and their protein properties

The characteristic 14-3-3 domain generated by hmmemit from HMM profile (PF00244) identified a total of 13, 6 and 33 14-3-3 protein sequences in foxtail millet (Si14-3-3), sorghum (Sb14-3-3) and maize (Zm14-3-3). Identification of respective *14-3-3* encoding genes and removal of alternate transcripts showed the presence of 8, 5 and 26 gene family members in foxtail millet (*Si14-3-3_a* to *Si14-3-3_h*), sorghum (*Sb14-3-3_a* to *Sb14-3-3_e*), and maize (*Zm14-3-3_a* to *Zm14-3-3_z*), respectively (S2 Table). Of 8 *Si14-3-3* genes, 4 genes were found to encode alternate transcripts, whereas in case of sorghum, *Sb14-3-3_c* encoded for a single alternate transcript. Among *Zm14-3-3* genes, 12 were found to encode for 21 alternate transcripts, with a maximum of three alternate transcripts by *Zm14-3-3_f* and *Zm14-3-3_m*.

HMMSCAN confirmed the presence of characteristic 14-3-3 domain in all the proteins (S3 Table). The analysis also identified the presence of additional domains in Zm14-3-3 proteins. Particularly, Zm14-3-3_d, Zm14-3-3_j, Zm14-3-3_u and Zm14-3-3_v have an additional WCOR413, whereas Zm14-3-3_d and Zm14-3-3_y have a thylakoid-soluble phosphoprotein (TSP9) domain. Zm14-3-3_k has additional zinc finger (zf) domains including zf-C3HC4, zf-C3HC4_2, zf-C3HC4_3, zf-rbx1, zf-RING_2 and zf-RING_5. Zm14-3-3_n, Zm14-3-3_w, Zm14-3-3_x and Zm14-3-3_z possessed F-box, calreticulin, TIM and RRM domains, respectively (S3 Table). Further, motif compositions of 14-3-3 proteins were analyzed using MEME tool and 8 conserved motifs were identified across C_4_ panicoid genomes (S4 Table). The high degree of sequence conservation between 14-3-3 proteins of three species reveals the functional equivalence of these proteins.

The physicochemical properties of 14-3-3 proteins of sorghum, maize and foxtail millet were extensively analysed. The average protein length for Sb14-3-3 and Si14-3-3 was ~255 amino acids with a mean molecular weight of ~28 kDa. Conversely, length of Zm14-3-3 proteins varied drastically from 118 (Zm14-3-3_f) to 900 amino acids (Zm14-3-3_z) (S2 Table). The molecular weights also differed accordingly, ranging from 13.6 kDa for Zm14-3-3_f to 97.5 kDa for Zm14-3-3_z. The isoelectric points of Sb14-3-3 and Si14-3-3 proteins were also evidenced to be similar ranging from 4.7 to 5.1, whereas, isoelectric points of Zm14-3-3 proteins ranged from 4.7 to 9.7. The protein instability index revealed that 2 proteins from both Sb14-3-3 (Sb14-3-3_a and Sb14-3-3_e) and Si14-3-3 (Si14-3-3_d and Si14-3-3_h) were stable, while rest of the proteins were unstable. In case of Zm14-3-3, five proteins namely Zm14-3-3_g, Zm14-3-3_h, Zm14-3-3_l, Zm14-3-3_s and Zm14-3-3_w were stable (S2 Table). Most of 14-3-3 proteins of all three crop species were found to comprise aliphatic amino acids and aliphatic index reached an average of 85.3, 83 and 85 for sorghum, maize and foxtail, respectively. The hydropathicity values of all members of Sb14-3-3, Zm14-3-3 and Si14-3-3 proteins were less than zero, suggesting that these proteins were hydrophilic (S2 Table).

### Chromosomal distribution, gene duplication and structure of *14-3-3* genes

**Fig 1:**
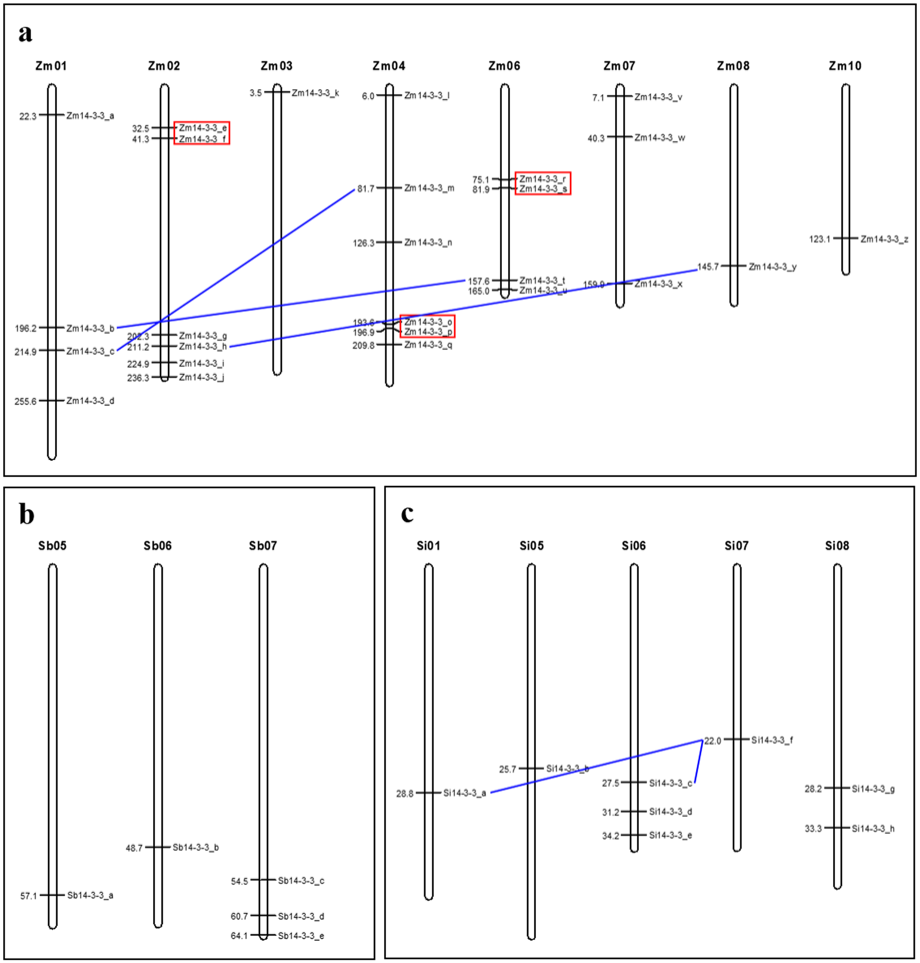
Chromosomal distribution and duplication of *14-3-3* genes. **a** Maize. **b** Sorghum. **c** Foxtail millet. Vertical bars represent chromosomes and the values at left indicate the position of genes in megabase. Genes are marked in the right and Zm, Sb and Si denotes *Zea mays*, *Sorghum bicolor* and *Setaria italica*, respectively. Lines denote segmental duplication, whereas tandemly duplicated genes are highlighted in boxes.

Physical mapping of identified *14-3-3* genes onto respective crop genomes shows the distribution of the genes across chromosomes (Fig 1; S2 Table). In foxtail millet, *14-3-3* genes were present in chromosomes 1, 5, 6, 7, 8 and 9 with a maximum of three *14-3-3* genes on chromosome 6, two on chromosome 8 and one each on chromosomes 1, 5 and 7 (Fig 1). Of the 5 *Sb14-3-3* genes, three were located on chromosome 7 and one each on chromosome 5 and 6. In case of maize, 26 *Zm14-3-3* genes were found to be unequally distributed on all the chromosomes except chromosome 5 and 9. Maize chromosomes 2 and 4 have maximum number of 6 *14-3-3* genes each, followed by chromosomes 1 and 4 with four *14-3-3* genes each. Three *14-3-3* genes were found to be encoded on maize chromosome 7 whereas chromosomes 3, 8 and 10 had one *14-3-3* gene each.

The two prime events of gene duplication namely tandem and segmental occurred within the genome resulted in more copies of genes belonging to the same family. To ensure this, the mechanisms involved in expansion of *14-3-3* members in these three crop species were examined. The analysis revealed that none of *Sb14-3-3* genes were duplicated, whereas three *14-3-3* gene-pairs of maize underwent both segmental and tandem duplication. Three genes in foxtail millet were found to be segmentally duplicated (Fig 1; S5 Table). It shows that the lesser number of *14-3-3* genes in sorghum might be due to the absence of gene duplication, but both segmental and tandem duplication played a prominent role in expansion of *14-3-3* gene family in maize. In case of foxtail millet, segmental duplication has a minimal role in expansion of *14-3-3* gene family in its genome.

Analysis of gene structures showed the differential distribution of intronic regions amid the exonic sequences across *14-3-3* genes of all the three crop species (S1 Fig). Out of 8 *Si14-3-3* genes, three had 4 introns and two had 3 introns, whereas *Si14-3-3_h* had the maximum number of 6 introns. *Sb14-3-3_a* and *Sb14-3-3_e* have 5 and 3 introns, respectively, while *Sb14-3-3_b*, *Sb14-3-3_c* and *Sb14-3-3_d* have 4 introns each. *Zm14-3-3_f* and *Zm14-3-3_n* have a minimum of 1 and 2 introns, respectively. A maximum of 20 introns were evidenced in *Zm14-3-3_y*. Noteworthy, 10 *Zm14-3-3* genes have 4 introns each. Further, variation in gene lengths was observed with respect to intron-exon distribution. The size of *Si14-3-3* genes ranged from 1643 (*Si14-3-3_a*) to 7582 (*Si14-3-3_h*). In case of sorghum and maize millet, gene sizes ranged from 3157 bp (*Sb14-3-3_c*) to 5753 bp (*Sb14-3-3_a*) and 1175 bp (*Zm14-3-3_x*) to 13512 bp (*Zm14-3-3_u*), respectively.

### Multiple sequence alignment and phylogenetic analysis of *14-3-3* genes

**Fig 2:**
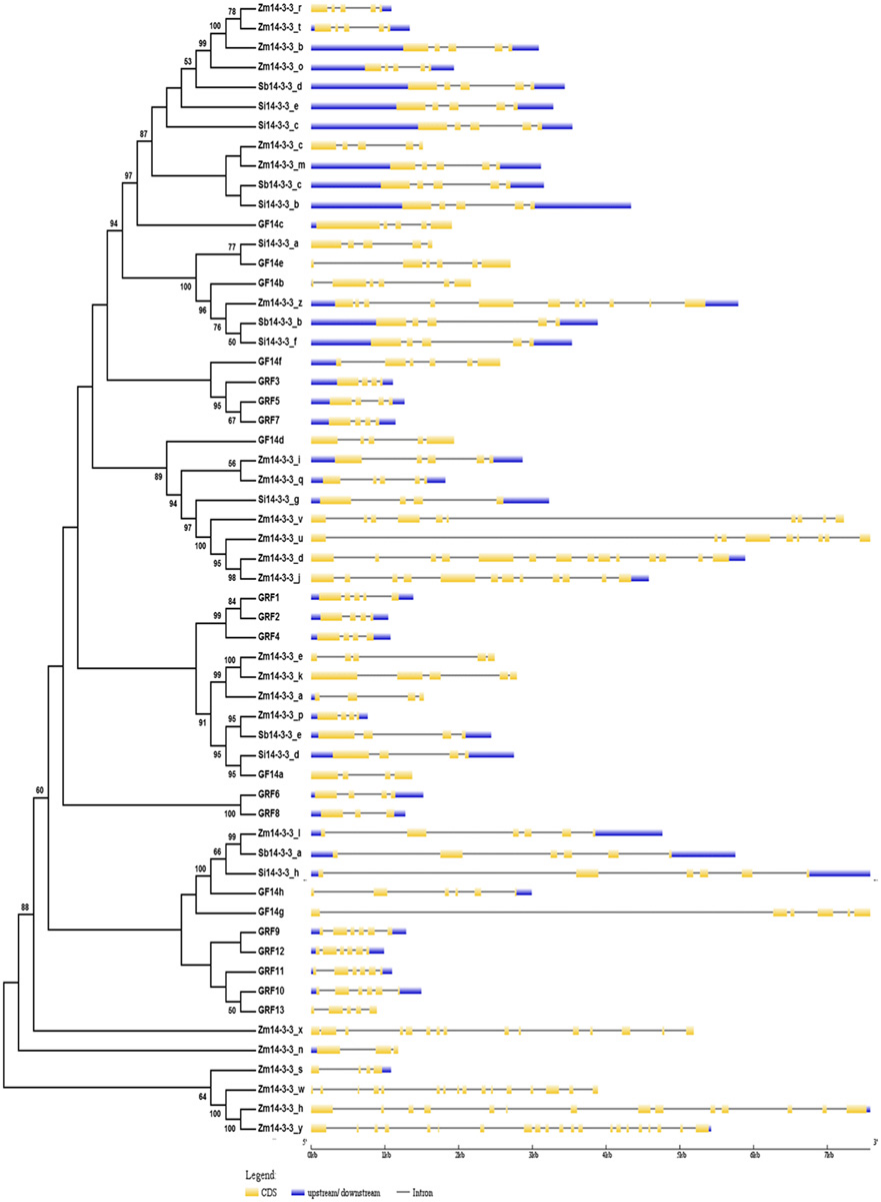
Phylogenetic tree and gene organization of 14-3-3 proteins. The unrooted phylogenetic tree was constructed with maize 14-3-3 proteins from sorghum (Sb14-3-3), maize (Zm14-3-3), foxtail millet (Si14-3-3), *Arabidopsis* (GRF) and rice (GF). The intron-exon positions of respective members are shown in the right.

A multiple sequence alignment was performed using 14-3-3 protein sequences of sorghum, maize, foxtail millet, rice and *Arabidopsis*. The alignment indicated that 14-3-3 amino acid sequences of sorghum, maize and foxtail millet are highly conserved except at N-terminal and C-terminal regions (S2 Fig). This observation conforms to the previous report on soybean *14-3-3* gene family [3]. The same set of 14-3-3 sequences was used for construction of phylogenetic tree by neighbour-joining (NJ) method (Fig 2). Instead of showing two distinct evolutionary groups, namely Non-Epsilon and Epsilon group, phylogenetic tree displayed a scattered pattern of Non-Epsilon and Epsilon group members (Fig 2), which could be plausibly due to the high percentage of sequence divergence among the investigated genomes.

### Functional annotation of 14-3-3 proteins

The putative participation of 14-3-3 proteins in diverse biological processes and molecular functions was revealed through gene ontology annotation (S3 Fig; S6 Table). A maximum of these proteins were predicted to be involved in 5 biological processes namely, primary and cellular metabolic processes, response to chemical stimulus, organic substance embolic and catabolic processes. This supports the potential of 14-3-3 proteins as one of the master regulators of central metabolism. Molecular function of 14-3-3 proteins revealed that these proteins were capable of binding, catalytic activity and enzyme regulator activity (S3 Fig), whereas cellular component analysis showed the localization of majority of 14-3-3 proteins in intracellular organelles and cytoplasm (S3 Fig). The presence of 14-3-3 proteins in ubiquitin ligase complex is also shown. A few 14-3-3 proteins were also predicted to be localized in cell wall and thylakoids.

### Expression profiling of *14-3-3* genes in different tissues of C_4_ panicoids

**Fig 3:**
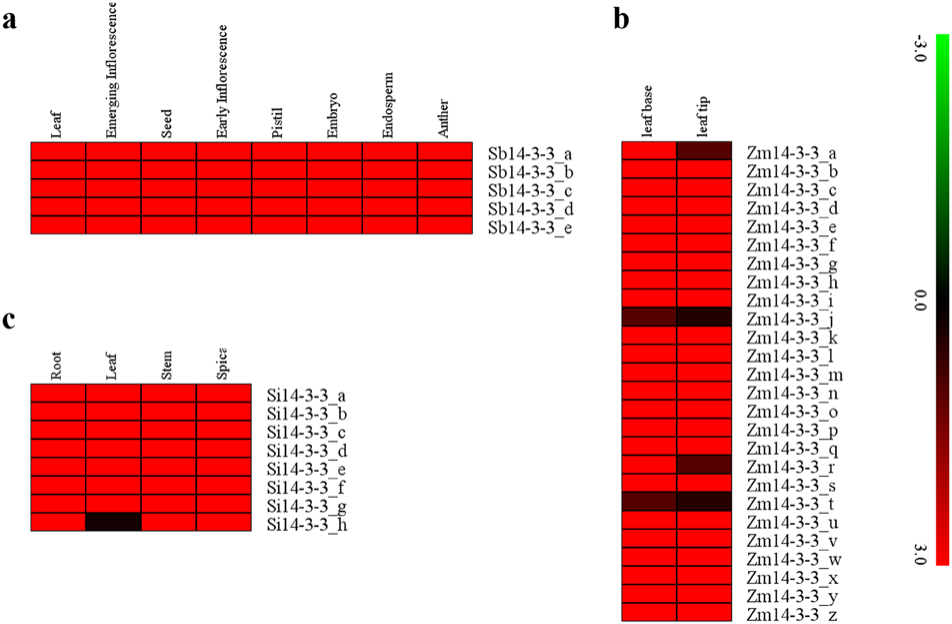
Tissue-specific expression profiles of *14-3-3* genes. **a** Sorghum. **b** Maize. **c** Foxtail millet. Illumina RNA-seq data of different tissues of these crops were re-analyzed and heat map was generated. Bar at the right represents log_2_ transformed values, thereby values -3.0, 0.0 and 3.0 represent low, intermediate and high expression, respectively.

The expression pattern of *Si14-3-3*, *Sb14-3-3* and *Zm14-3-3* genes were studied in 8 tissues of sorghum (leaf, emerging inflorescence, seed, early inflorescence, pistil, embryo, endosperm and anther), 2 tissues of maize (leaf base and leaf tip), and 4 tissues of foxtail millet (spica, stem, leaf and root), respectively. Predominant *14-3-3* genes were found to be highly expressed in all the tissues of respective plant species (Fig 3). All the genes except *Si14-3-3_h* of foxtail millet showed higher expression in its four tissues, whereas all 8 tissues of sorghum showed higher expression of all *Sb14-3-3* genes. In maize, higher expression of all *Zm14-3-3* genes except *Zm14-3-3_a*, *Zm14-3-3_j*, *Zm14-3-3_r* and *Zm14-3-3_t* was evidenced in leaf base and leaf tip. Tissue-specific higher expression of *Zm14-3-3_a* and *Zm14-3-3_r* was observed in leaf base of maize. Hence, this analysis demonstrated higher expression of *14-3-3* genes in almost all the test tissues of foxtail millet, sorghum and maize.

### Comparative mapping of *Si14-3-3* genes with C_4_ and C_3_ genomes of Poaceae

**Fig 4:**
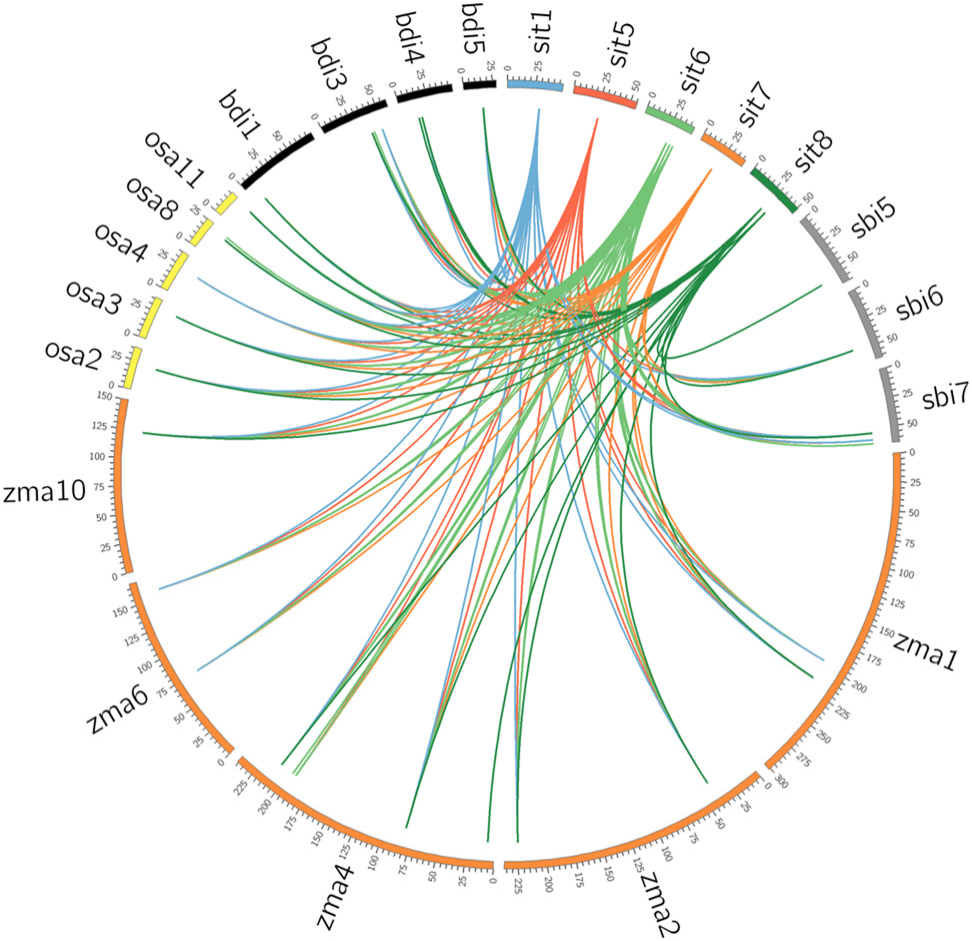
Comparative physical mapping of *14-3-3* genes. Orthologous relationship of foxtail millet *14-3-3* genes (Sit) with the genes of sorghum (Sbi), maize (Zma), rice (Osa) and *Brachypodium* (Bdi). The coloured blocks represent the chromosomes.

The orthologous relationship between *14-3-3* genes of foxtail millet and other Poaceae members including sorghum, maize, rice and *Brachypodium* was studied (Fig 4; S7 Table). Between foxtail millet and sorghum, *Si14-3-3_d* and *Si14-3-3_h* were found to be orthologous with *Sb14-3-3_e* and *Sb14-3-3_a*, respectively. Further, *Si14-3-3_e*, *Si14-3-3_f* and *Si14-3-3_g* showed considerable homology to *Sb14-3-3_d*. In case of foxtail millet-maize synteny, *Si14-3-3_a*, *Si14-3-3_d*, *Si14-3-3_e*, *Si14-3-3_g* and *Si14-3-3_h* showed significant orthology to *Zm14-3-3_f*, *Zm14-3-3_p*, *Zm14-3-3_m*, *Zm14-3-3_i* and *Zm14-3-3_l* respectively. Orthologous relationship between foxtail millet and rice showed that *Si14-3-3_a*, *Si14-3-3_e*, *Si14-3-3_f* and *Si14-3-3_g* revealed higher similarity to *GF14b* of rice. Foxtail millet and *Brachypodium 14-3-3* based comparative mapping showed that 5 *Si14-3-3* genes namely *Si14-3-3_a*, *Si14-3-3_b*, *Si14-3-3_d*, *Si14-3-3_g* and *Si14-3-3_h* were syntenic towards *Brachypodium* genes *Bradi5g12510*, *Bradi3g36480*, *Bradi3g38640*, *Bradi4g16640* and *Bradi4g13970*, respectively.

### Duplication and divergence rate of paralogs and orthologs

The effect of Darwinian positive selection in duplication and divergence of *14-3-3* genes was analysed by estimating the ratios of non-synonymous (Ka) versus synonymous (Ks) substitution (Ka/Ks) following Mishra et al. [25]. The Ka/Ks ratio for tandemly duplicated maize gene pairs ranged from 0.08 to 0.1, whereas Ka/Ks ratios of segmentally duplicated gene pairs ranged from 0.09 to 0.1 for maize and 0.06 to 0.09 for foxtail millet. This suggested that the duplicated *14-3-3* genes were under strong purifying selection pressure (Ka/Ks <1) (S5 Table). The duplication events were predicted to occur ~9 MYA in maize and ~18 MYA in foxtail millet. This conforms to the whole genome duplication events occurred in maize and foxtail millet genomes ~5–12 MYA and ~18 MYA, respectively [31,32]. Among the *14-3-3* orthologs of foxtail millet and other Poaceae members, the average Ka/Ks ratio was maximum between foxtail millet and *Brachypodium* (0.81), followed by rice (0.48), maize and sorghum (0.27 each). The relatively higher rate of synonymous substitution between foxtail millet and *Brachypodium* indicated their earlier divergence ~62 MYA (S7 Table), whereas lower Ka/Ks rate between foxtail millet–maize and—sorghum revealed their recent divergence ~20 MYA [32].

### Expression profiles of *Si14-3-3* genes during abiotic stresses and hormonal treatments

**Fig 5:**
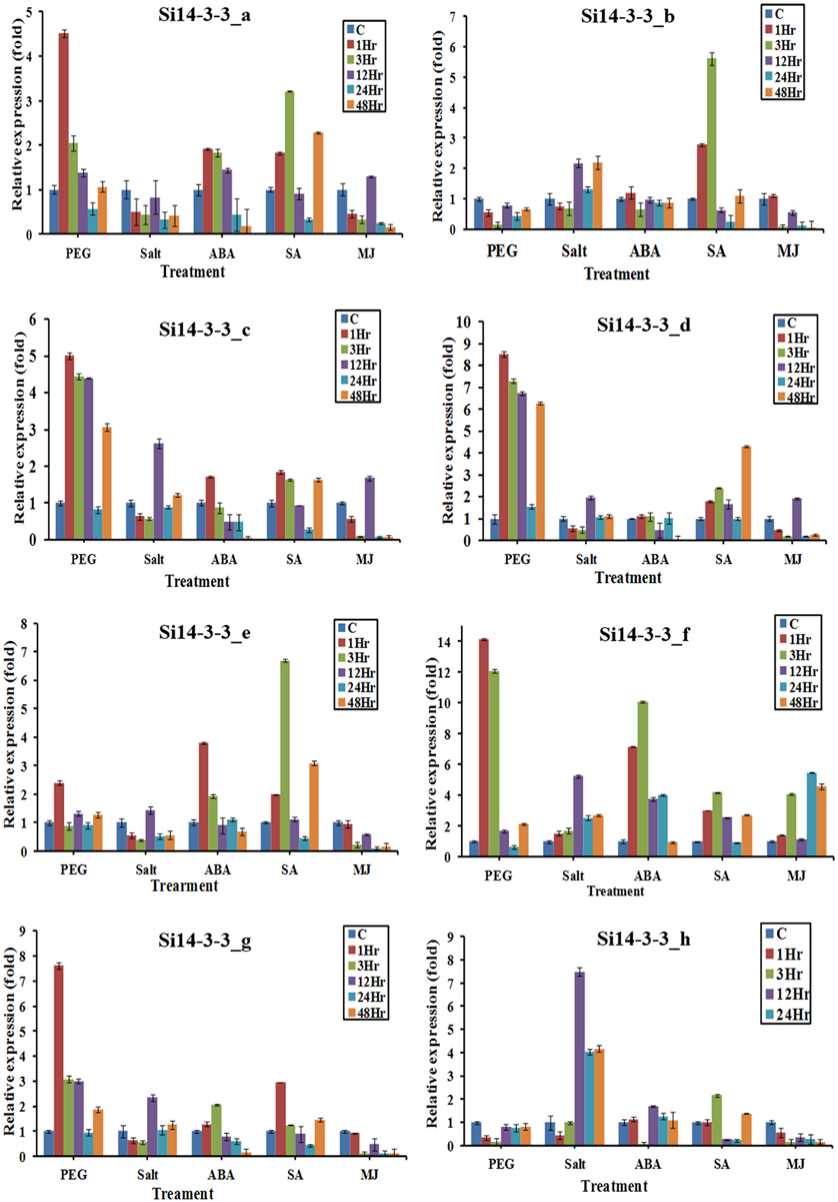
Expression profiles of *Si14-3-3* genes during abiotic stresses and hormone treatments. Relative expression pattern of *Si14-3-3* genes analyzed using qRT-PCR under dehydration stress (PEG), salt stress, ABA treatment, salicylic acid (SA) treatment and methyl jasmonate (MJ) treatment, for 0, 1, 3, 12, 24 and 48 hr. The relative expression ratio of each gene was calculated relative to its expression in control sample (0 hr). Act2 was used as an internal control to normalize the data. The error bars representing standard deviation were calculated based on three technical replicates for each biological duplicates.

Expression profiling of all eight *Si14-3-3* genes during stress and hormonal treatments showed that these genes have exerted variations in their expression patterns (Fig 5). During early phase of PEG treatment (drought stress), a relatively higher expression of *Si14-3-3_a*, *Si14-3-3_c*, *Si14-3-3_d*, *Si14-3-3_f* and *Si14-3-3_g* was observed. Conversely, *Si14-3-3_b* and *Si14-3-3_g* showed no notable change in fold expression but both the genes were found to be significantly down-regulated at 3^rd^ hr of salinity stress (Fig 5). The expression profiles of all *Si14-3-3* genes were almost similar during salinity stress except *Si14-3-3_c* and *Si14-3-3_h* which showed a considerable higher expression at 12^th^ hr of salt stress. In case of abscisic acid treatment, *Si14-3-3_e* and *Si14-3-3_f* were found to be up-regulated considerably at the early phase while other genes did not show any notable change in their expression patterns throughout the treatment. Salicylic acid induced higher expression of *Si14-3-3_a*, *Si14-3-3_b* and *Si14-3-3_e*, whereas methyl jasmonate (MJ) treatment showed no considerable up-regulation of *Si14-3-3* genes. On contrary, all the genes except *Si14-3-3_f* were down-regulated at late phase of MJ treatment (Fig 5).

### Subcellular localization of Si14-3-3 proteins

**Fig 6:**
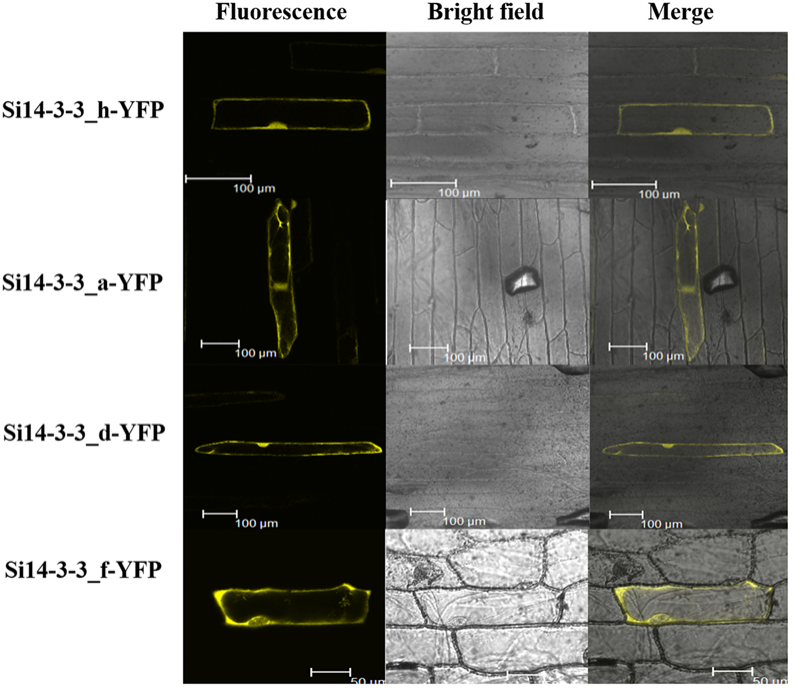
Subcellular localization of candidate Si14-3-3 proteins in onion epidermal cells. Onion epidermal cells were transiently transformed with constructs containing vector *pAMPAT-MCS*-14-3-3:YFP through particle bombardment method. Subcellular localization of respective Si14-3-3:YFP fusion proteins were viewed using fluorescent confocal microscope.

Isoform-specific cellular localization results showed differential localization of Si14-3-3_a, Si14-3-3_d, Si14-3-3_f and Si14-3-3_h proteins within the cell (Fig 6). Interestingly, Si14-3-3_f-YFP showed clear localization in cytoplasm as well as in nuclear membrane whereas other three members were evenly distributed throughout cell without any subcellular eccentricity.

### Novel interaction between Si14-3-3_f and SiRSZ21A

**Fig 7:**
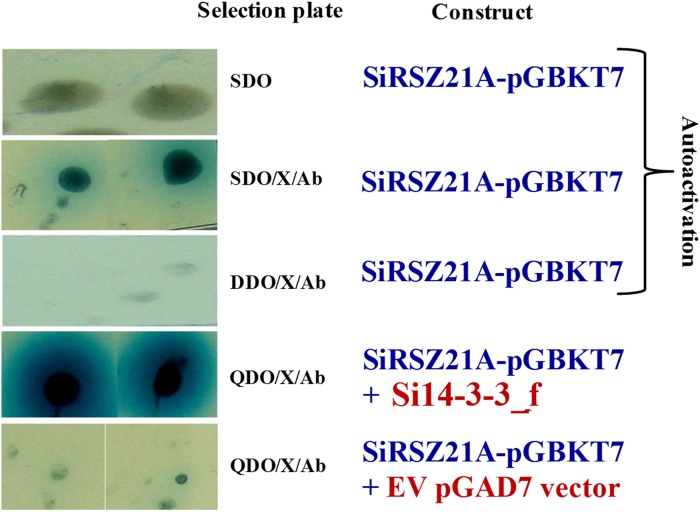
Yeast two-hybrid assay between Si14-3-3_f and SiRSZ21A. Y2H gold was co-transformed with both the constructs. Interaction was confirmed by culturing the co-transformants on selective media. Empty-BD constructs were used against the SiRSZ21A as controls. Autoactivation was also checked for SiRSZ21A.

**Fig 8:**
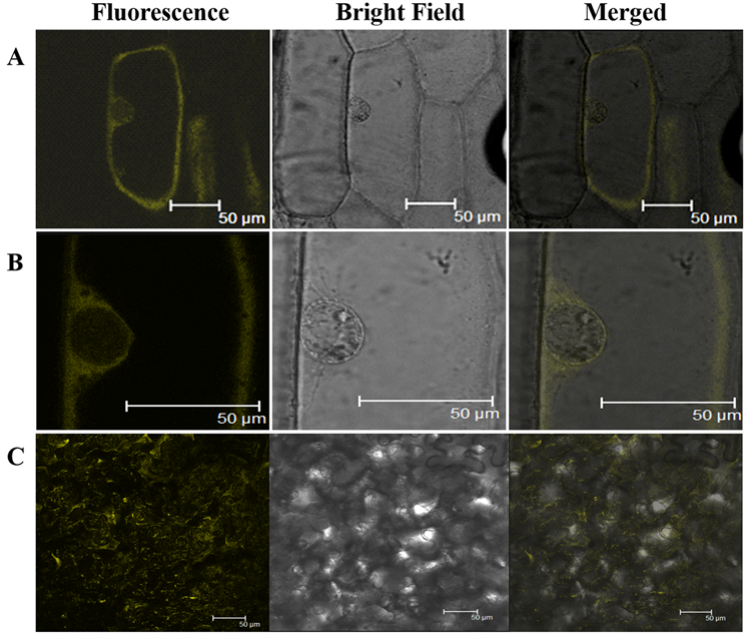
*In vivo* BiFC analysis for confirming interaction of Si14-3-3_f protein with SiRSZ21A splicing factor. **A** whole cell, **B** nucleus, shows the interaction in onion epidermal cells. **C.** interaction in *N*. *benthamiana* leaf.

**Fig 9:**
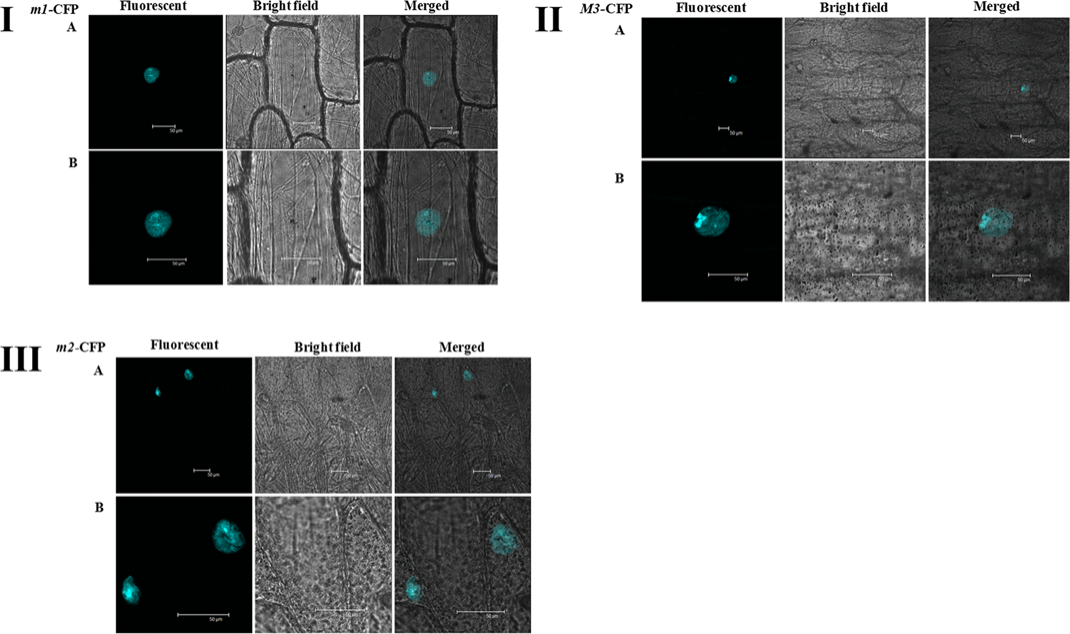
Sub-cellular localization of mutants showing nucleus localization. **I** *m1*, **II** *m2*, **III** *m3*; **A** whole cell, **B** magnified view of the nucleus. More accumulation was seen in speckle region.

**Fig 10:**
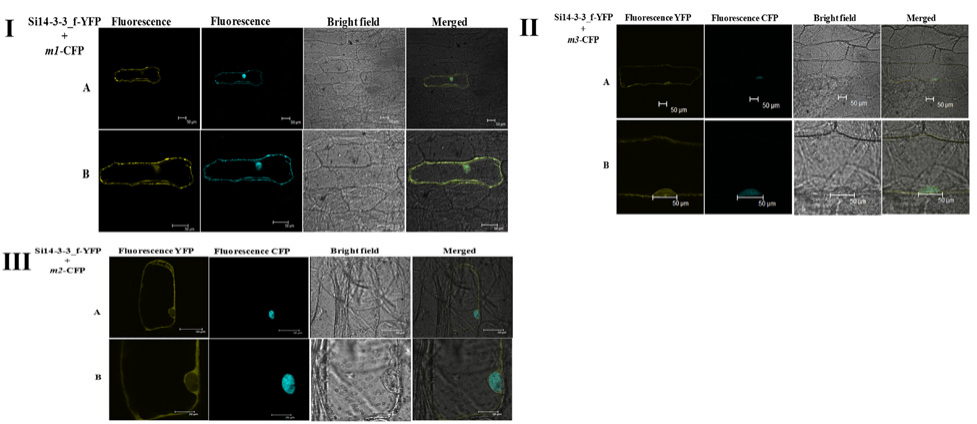
Si14-3-3 and mutant SiRSZ21A (*m2* and *m3*) co-expressed in onion epidermal cells. Localization of m2 in nucleus is evidenced. **A** whole cell, **B** magnified view of nucleus.

14-3-3 binding site in foxtail millet homologue (*SiRSZ21A*) of *Arabidopsis thaliana* nucleocytoplasmic shuttling phosphoprotein (AtRSZp22) showed the presence of three serine residues (RRRSR**S**R**S**RSR**S**P), which could get phosphorylated to act as binding site for Si14-3-3_f protein. One-on-one interaction performed using yeast two-hybrid assay showed the interaction between Si14-3-3_f and SiRSZ21A proteins (Fig 7) and the interaction was confirmed *in vivo* with bimolecular fluorescence complementation assay in onion epidermis and *Nicotiana benthamiana* leaf (Fig 8). Of note, this interaction was seen in cytoplasmic region in the cells. To validate the involvement of serine residues in the interaction with Si14-3-3_f, serine residues are substituted with alanine [*m1* (S120→A120), *m2* (S122→A122), *m3* (S124→A124)]. These SiRSZ21A mutants showed nuclear localization (Fig 9), whereas co-localization study revealed that m1-CFP was distributed throughout cell in the presence of Si14-3-3_f (Fig 10). In contrast, m2-CFP and m3-CFP were concentrated in nucleus even in the presence of S14-3-3_f (Fig 10).

## Discussion

14-3-3 proteins are important regulatory proteins found ubiquitously in all eukaryotes and are reported to function in several physiological, morphological and stress-responsive pathways in plants. In spite of its importance, these proteins were studied to a lesser extent and among C_4_ panicoids, no comprehensive investigation has been performed. Considering this, the present study attempted to characterize 14-3-3 proteins of C_4_ panicoids with emphasis on foxtail millet (*S*. *italica*), which is now a model plant for studying systems biology of millets, cereals and bioenergy grasses [17,18]. The availability of draft genome sequences of major crop plants facilitated *in silico* identification of *14-3-3* gene family members and their further characterization. To date, genome-wide analyses of *14-3-3* genes have been performed in *Arabidopsis* (13 genes) [6,7], rice (8 genes) [8], barley (5 genes) [9], tobacco (17 genes) [10], cotton (6 genes) [11] and soybean (18 genes) [3]. Though the number of *14-3-3* genes identified in foxtail millet and sorghum (8 and 5, respectively) are in accordance with the numbers identified in related species, the number predicted in maize is first ever identified highest number of *14-3-3* genes. Maize genome underwent a considerable expansion (to 2.3 gigabases) ~3 million years ago (MYA) through proliferation of long terminal repeat retrotransposons [31], and this could be the plausible reason behind the presence of multiple copies of *14-3-3* genes in maize genome. In this context, the number of *14-3-3* genes across the sequenced plant genomes was analyzed (S4 Fig). Among Poaceae members, maize has the highest number of *14-3-3* genes (26), followed by rice and foxtail millet (8 each), then *Brachypodium* with 7 *14-3-3* members and least being sorghum (5 genes). Of note, the number of *14-3-3* genes in sorghum is least among all land plants. The analysis of gene structures of *Sb14-3-3*, *Zm14-3-3* and *Si14-3-3* genes showed a diverse distribution of introns and exons, which indicate the evolutionary changes that have occurred in respective genomes.

Domain analysis showed the presence of additional domains in some Zm14-3-3 proteins. Zm14-3-3_d, Zm14-3-3_j, Zm14-3-3_u and Zm14-3-3_v have an additional WCOR413 domain which is reported to have a role in cold acclimation [33]. Zm14-3-3_d and Zm14-3-3_y have a thylakoid-soluble phosphoprotein (TSP9) domain which is a plant specific protein. TSP9 is phosphorylated and released in response to changing light conditions from photosynthetic membrane [34]. Noteworthy, Zm14-3-3_k has additional zinc finger (zf) domains including zf-C3HC4, zf-C3HC4_2, zf-C3HC4_3, zf-rbx1, zf-RING_2 and zf-RING_5. F-box, calreticulin, TIM and RRM domains were found to be present in Zm14-3-3_n, Zm14-3-3_w, Zm14-3-3_x and Zm14-3-3_z, respectively.

F-box domain was first reported in cyclin F. These domains comprise of 50 amino acids which play vital roles in mediating protein-protein interaction by binding with SCF protein Skp1 [35]. Calreticulin is a high-capacity calcium-binding domain [36]. TIM is triosephosphate isomerase domain which mediates the conversion of glyceraldehyde-3-phosphate to dihydroxyacetone phosphate and vice versa. These domain containing proteins are reported to involve in various metabolic pathways [37]. RRM is RNA recognition motif which binds to single-stranded RNAs as well as few proteins [38].

Instead of showing two distinct evolutionary groups, namely Non-Epsilon and Epsilon group, phylogenetic tree displayed a scattered pattern of Non-Epsilon and Epsilon group members. According to DeLille et al. [7], Non- Epsilon members will have 4 exons and 3 introns and in some cases an extra intron will be present in 5’ leader, whereas Epsilon members possess 6–7 exons and 4–6 introns. This group-based phylogenetic tree was reported in *Arabidopsis* [6,7], rice [8] and soybean [3], but in C_4_ panicoid grasses it could not be deduced (Fig 2). This could be plausibly due to high percentage of sequence divergence among the genomes.

Functional annotation of 14-3-3 proteins of sorghum, maize and foxtail millet indicated their diverse roles in various biological and molecular functions. Predominant 14-3-3 proteins were shown to be involved in metabolic processes. Recently, Diaz et al. [39] performed a combinatorial analysis of metabolites and enzyme activities in *14-3-3* overexpression and knock-out *Arabidopsis* plants with protein-protein interactions, and found that 14-3-3 proteins are crucial for a majority of metabolic pathways. Molecular function of 14-3-3 proteins revealed that these proteins were capable of binding, catalytic activity and enzyme regulator activity (S3 Fig). Binding is crucial for 14-3-3 proteins since these proteins execute their biological roles through direct protein-protein interactions [40], which includes two main features, (i) binding of phosphoserine or phosphothreonine residue containing motifs of target proteins with conserved amphipathic groove of 14-3-3 protein, and (ii) binding of 14-3-3 protein with the target. However, this interaction is affected by factors such as absolute activity, interaction of 14-3-3 with other proteins and localization of 14-3-3 proteins in the cell [40]. Cellular component analysis also revealed the distribution of 14-3-3 proteins at almost all the cellular organelles, which is in accordance to reports on localization of 14-3-3 family members inside organelles including chloroplasts [41], nucleus [42], mitochondria [43] and cytoplasm [42]. This demonstrates the global regulatory potential of 14-3-3 proteins and its necessity for variations in expression and function [7].

Comparative mapping of *Si14-3-3* genes in C_3_ and C_4_ genomes demonstrated that *14-3-3* genes were conserved across C_3_ and C_4_ genomes. Although similar genome-wide studies on important gene families such as *NAC* [28], *WD40* [25], *DCL*, *AGO*, *RDR* [30], *MYB* [17] and *C*_*2*_ *H*_*2*_ *zinc fingers* [26] in foxtail millet reported a decrease in synteny from sorghum, maize, rice and *Brachypodium*, the present study observed five *Si14-3-3* orthologs in all four crops, of which *Si14-3-3_g* was evidenced to be highly conserved across these genomes. This substantiates that large parts of all 14-3-3 proteins, irrespective of the organisms, are evolutionarily conserved [4].

Expression profiling of all the eight *14-3-3* genes (*Si14-3-3_a* to *Si14-3-3_h*) during different abiotic stresses and hormonal treatments at different time-points showed a dynamic expression pattern of these genes. This differential expression pattern supports the fact that these *Si14-3-3* genes are involved in the regulation of complex metabolic as well as stress responsive pathways for normal growth and stress acclimation, respectively. The present observations accord to the recent report in cotton, where it was suggested that the stress treatments considerably enhanced the altered expression of *14-3-3* genes [44]. Since no reports were available on the study of *14-3-3* expression patterns during stress in foxtail millet, this expression data would serve as a base for conducting further studies to understand the roles of *14-3-3* genes in imparting stress tolerance in foxtail millet.

It was reported that 14-3-3 proteins subcellular localization within a cell could be affected by specific target proteins rather than its own intrinsic property [45]. Sub-cellular localization of Si14-3-3 proteins showed differential localization of Si14-3-3_a, Si14-3-3_d, Si14-3-3_f and Si14-3-3_h proteins within the cell, whereas Si14-3-3_f-YFP showed clear localization in both cytoplasm and nuclear membrane. These results indicate that different isoforms have different targets and varied affinity to proteins, as supported by Si14-3-3_f which showed different location. Although this provides evidence that some members might have target specificity, it might also be possible that in the absence of one member, another can complement its function to an extent.

Reports have demonstrated that phosphorylation of RS domains regulates nucleocytoplasmic shuttling of splicing factors [46–48]. Being a known interacting agent with phosphorylated proteins, 14-3-3 proteins could be involved in this process. To decipher this, *Arabidopsis thaliana* RSZp22, a nucleocytoplasmic shuttling phosphoprotein was chosen [48] and its foxtail millet homologue was identified (*SiRSZ21A*). 14-3-3 binding site identification in SiRSZ21A protein using MotifScan analysis predicted three serine residues (RRRSR**S**R**S**RSR**S**P), which could get phosphorylated to act as binding site for Si14-3-3_f protein. Interestingly, it was observed that this motif was present within RS domain of SiRSZ21A. Since, earlier report by Colwill et al. [24] has demonstrated that RS domain of SR protein is responsible for its sub-cellular localization, one-on-one interaction has been performed using yeast two-hybrid assay which showed the occurrence of interaction between Si14-3-3_f and SiRSZ21A proteins in cytoplasmic region. To confirm the involvement of serine residues in the interaction with Si14-3-3_f, serine residues are substituted with alanine [*m1* (S120→A120), *m2* (S122→A122), *m3* (S124→A124)]. These SiRSZ21A mutants showed nuclear localization, whereas co-localization study revealed that m1-CFP was distributed throughout the cell in the presence of Si14-3-3_f. In contrast, m2-CFP and m3-CFP were concentrated in nucleus even in the presence of S14-3-3_f (Fig 2). These observations suggest that residue S118 in SiRSZ21A (*m1*) was either unphosphorylated or its phosphorylation is not affecting the interaction with Si14-3-3_f. Conversely, S120 and S124 residues might be phosphorylated resulting in the occurrence of Si14-3-3_f interaction. SiRSZ21A-GFP (*m1*) was distributed throughout the cell with Si14-3-3_f, while m2 and m3 were completely concentrated in the nucleus. This signifies that phosphorylation of S122 and/or S124 is completely blocked when serine is changed into alanine, subsequently resulting in loss of binding capacity. These results also prove that phosphorylation of a single serine residue (S122 or S124) is sufficient for interaction and phosphorylation of both serine residues phosphorylation might strengthen the interaction.

**Fig 11:**
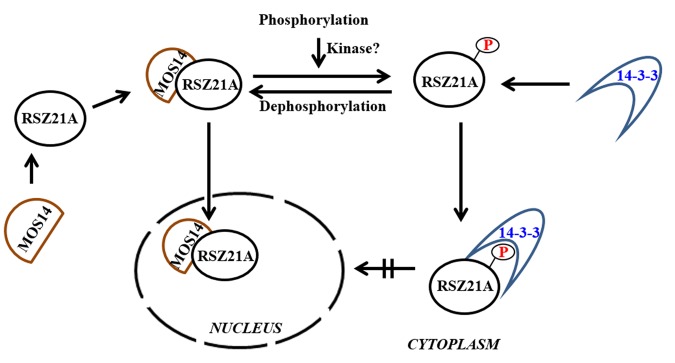
A model based on phosphorylation of S122 and/or S124 in 14-3-3 for deciding the cellular location of SiRSZ21A. When phosphorylation of SiRSZ21A was prevented, 14-3-3 proteins could not bind to SiRSZ21A and therefore SiRSZ21A was localized in nucleus. Another possibility for cytoplasmic retention of SiRSZ21A may be due to the masking effect of 14-3-3 on MOS14 binding site in phosphorylated SiRSZ21A.

RS domain containing proteins are mobile proteins which can move out of the nucleus during the change in phosphorylation condition or tethered with mRNA [46,47]. Conversely, another study showed that AtRSZ21 is transported into nucleus with the help of MOS14 [49]. Though these reports suggest the mobility mechanism of RS proteins between nucleus and cytoplasm, the present study demonstrates that phosphorylation play a key role in cytoplasmic retention of SiRSZ21A through its interaction with Si14-3-3_f. When phosphorylation of SiRSZ21A was prevented, 14-3-3 proteins could not bind to SiRSZ21A and therefore SiRSZ21A was localized in nucleus. It is also speculated that phosphorylation of serine residues may disrupt the interaction between SiRSZ21A and MOS14 and thus creates a binding site for 14-3-3 protein. The interaction between 14-3-3 and SiRSZ21A results in retention of SiRSZ21A in cytoplasm. Another possibility for cytoplasmic retention of SiRSZ21A may be due to the masking effect of 14-3-3 on MOS14 binding site in phosphorylated SiRSZ21A. Based on these, a model based on phosphorylation of S122 and/or S124 in 14-3-3 for deciding the cellular location of SiRSZ21A has been resolved (Fig 11).

The nucleo-cytoplasmic shuttling of RS domain containing proteins supports the diverse functions of these proteins in addition to mRNA splicing [50]. Therefore, considering the importance of SiRSZ21A splicing factor in different physiological and molecular aspects, it could be concluded that Si14-3-3_f might play a direct role in splicing events by regulating SiRSZ21A sub-cellular localization. Keeping this in view, further studies have been initiated to extrapolate the role of 14-3-3 proteins in splicing mechanism and standardization of silencing as well as over-expression in foxtail millet system is in progress. Taken together, the present study demonstrates that SiRSZ21A is a nucleo-cytoplasmic shuttling protein which is dependent on dynamic mechanism of phosphorylation/dephosphorylation and interaction of Si14-3-3_f, and it is concluded that Si14-3-3_f might play an indirect role in the splicing events by regulating the sub-cellular localization of SiRSZ21A. In addition, the comprehensive analysis of *14-3-3* gene family members in foxtail millet, sorghum and maize provides interesting information on their gene structures, protein domains, phylogenetic and evolutionary relationships, which could be useful in choosing candidate members for further functional characterization.

## Supporting Information

S1 Fig Gene structures of Sb14-3-3, Zm14-3-3 and Si14-3-3 proteins. Exons and introns are represented by green boxes and black lines, respectively.
(TIF)

S2 Fig Multiple sequence alignment of 14-3-3 protein sequences of sorghum (Sb), maize (Zm), foxtail millet (Si), rice (GF) and *Arabidopsis* (GRF). The conserved sequences are highlighted in black.
(TIF)

S3 Fig Gene Ontology annotation of Sb14-3-3, Zm14-3-3 and Si14-3-3 proteins. The Blast2GO output defining; **a** biological processes, **b** molecular functions, and **c** cellular components.
(TIF)

S4 Fig Distribution of *14-3-3* genes in sequenced plant genomes. (TIF)

S1 Table List of primers used in the present study. (DOC)

S2 Table Characteristic features of *14-3-3* gene family members identified in C_4_ panicoid crops. (XLS)

S3 Table Details of functional domains present in *14-3-3* genes of C_4_ panicoid crops. (XLS)

S4 Table Sequence logos for the conserved motifs of 14-3-3 proteins across the three C_4_ genomes. (XLS)

S5 Table Summary of tandemly and segmentally duplicated gene pairs and its evolutionary significance. (DOC)

S6 Table Gene Ontology annotation of 14-3-3 proteins. (XLS)

S7 Table Details of *Si14-3-3* orthologs in sorghum, maize, rice and *Brachypodium* and its evolutionary significance. (DOC)
