# Supplementary figures and images for: Unraveling 14-3-3 Proteins in C4 Panicoids with Emphasis on Model Plant Setaria italica Reveals Phosphorylation-Dependent Subcellular Localization of RS Splicing Factor
Source: PLoS One. 2015 Apr 7;10(4):e0123236. doi: 10.1371/journal.pone.0123236 (PMC4388342; doi:10.1371/journal.pone.0123236)

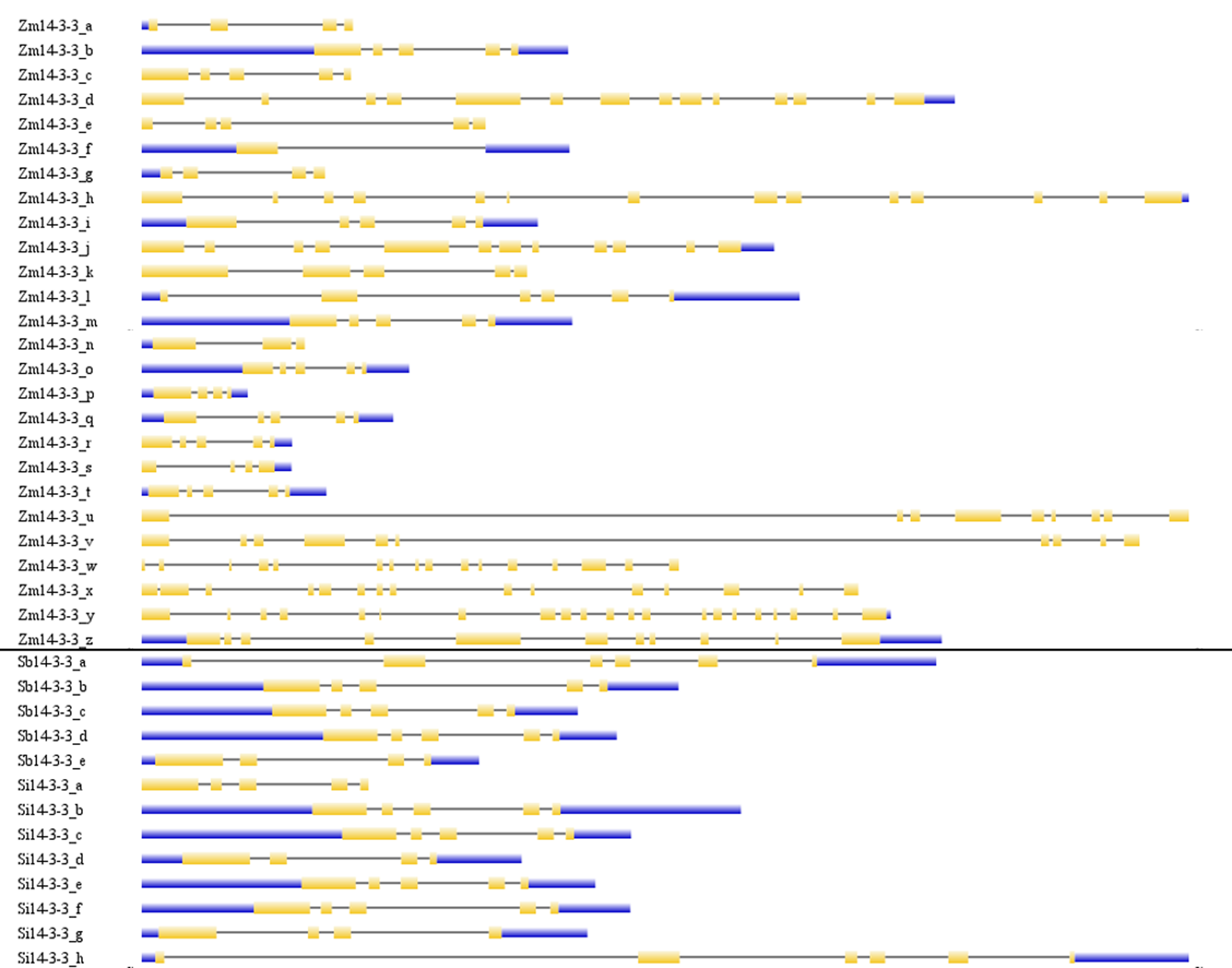

Supplement: S1 Fig — Exons and introns are represented by green boxes and black lines, respectively. (TIF) [file pone.0123236.s001.tif]

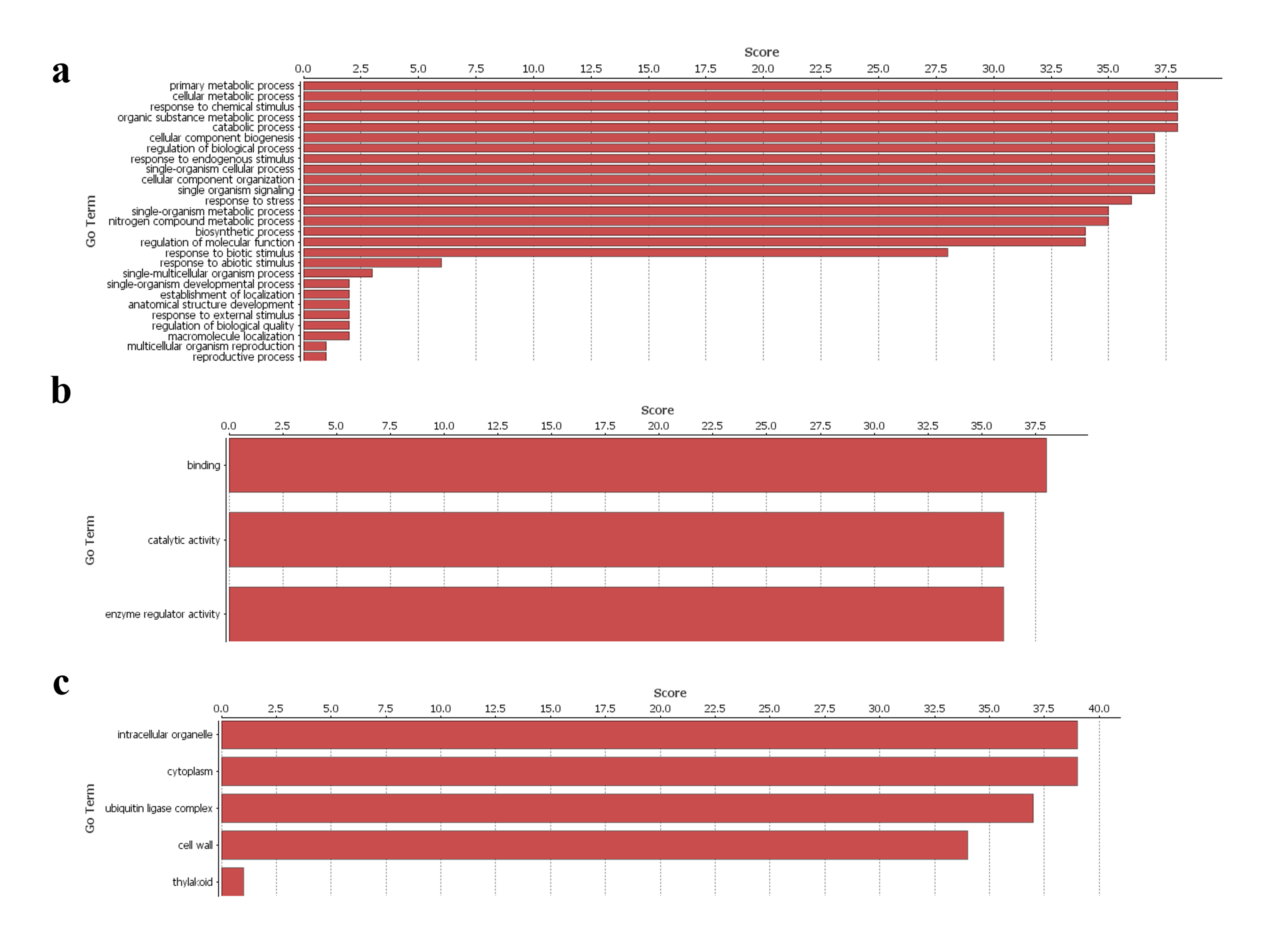

Supplement: S3 Fig — The Blast2GO output defining; a biological processes, b molecular functions, and c cellular components. (TIF) [file pone.0123236.s003.tif]

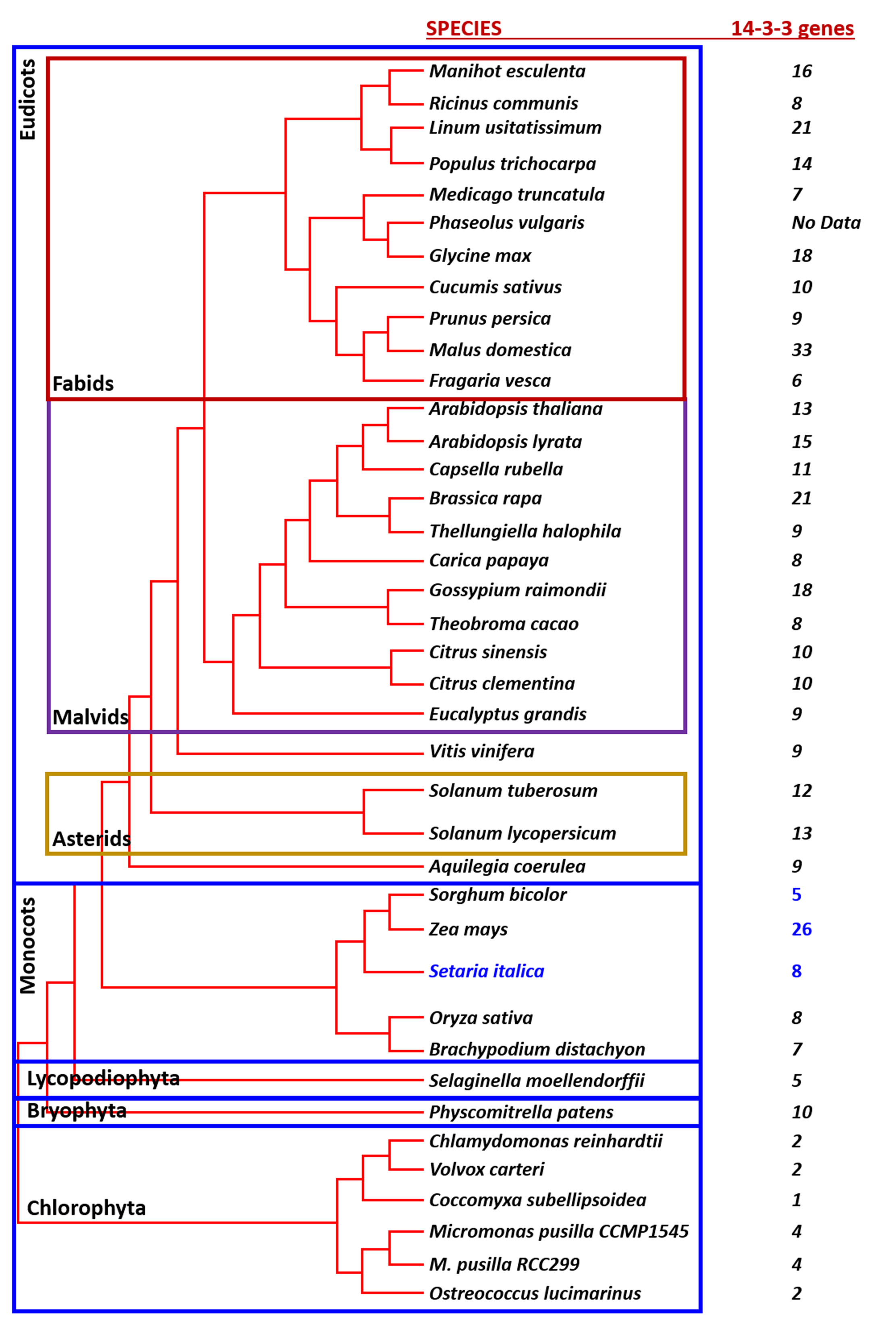

Supplement: S4 Fig — (TIF) [file pone.0123236.s004.tif]
