# Supplementary material for: Unraveling 14-3-3 Proteins in C4 Panicoids with Emphasis on Model Plant Setaria italica Reveals Phosphorylation-Dependent Subcellular Localization of RS Splicing Factor
Source: PLoS One. 2015 Apr 7;10(4):e0123236. doi: 10.1371/journal.pone.0123236 (PMC4388342; doi:10.1371/journal.pone.0123236)
Supplement: S1 Table — (DOC) [file pone.0123236.s005.doc]

**Table S1.** List of primers used in the present study.

| **GENE** | **FORWARD PRIMERS (5′- 3′)** | **REVERSE PRIMERS (5′- 3′)** |
| --- | --- | --- |
| **Quantitative Real-Time PCR analysis** | | |
| *Si14-3-3_a* | TCCATGTGCCCAGAAACTAC | TGGGAGTCTCCAATTTCCA |
| *Si14-3-3_b* | AGACAGCATAGCCGTGATTG | TGTAAGGCGTTGGGTTGTTA |
| *Si14-3-3_c* | CTTTGCATTGCCGCTAGTT | GACCACACACATGCTTGTCA |
| *Si14-3-3_d* | TGGGTAGTCGTGATGCTTGT | CTAACAGGGTTCCAGGAGGA |
| *Si14-3-3_e* | AGCCTGGTAAACCTTGATGG | CCGACCACTACCAGCCTACT |
| *Si14-3-3_f* | GCTGTAGCCGTGCTATGATT | CAAACCTGGCTCCAGTGAC |
| *Si14-3-3_g* | GAATCCGGAGAGGGTCAGTA | GAAATCCAGGAACATTGGCT |
| *Si14-3-3_h* | CGAACCCGTGTCCTCTAGTT | TGGTCACCAGTCCAGACACT |
| *Actin2* | CGCATATGTGGCTCTTGACT | GGGCACCTAAATCTCTCTGC |
| **Subcellular localization** | | |
| *Si14-3-3_a* | CTGCAGATGTCGCAGCCTGCTGAG | TCTAGACCCATCTCCAGACTCGCCCTTG |
| *Si14-3-3_d* | CCCAAGCTTATGGCGGCGGCAGGAGGAG | CGGAATTCCTCATCCTCGGGCTTGCTTG |
| *Si14-3-3_f* | CCCAAGCTTATGGCATCAGCAGAGCTTTC | CGGAATTCCTGCCCCTCGCTCGAGTCG |
| *Si14-3-3_h* | CGGAATTCATGGAGGAGCGGGAGAAGGTC | TCTAGAACCCTCCATGTCGATGTC |
| *SiRSZ21A* | CCAAGCTTATGGCCCGCTTGTACGTCG | CGGAATTCGCGCTCCTGCTGCGGCGGTACC |
| **Yeast two hybrid assay** | | |
| *Si14-3-3_f* | CATGGAGGCCGAATTCATGGCATCAGCAGAGCTTTC | GCAGGTCGACGGATCCCTGCCCCTCGCTCGAGTCGT |
| *SiRSZ21A* | CATGGAGGCCGAATTCATGGCCCGCTTGTACGTCG | CTGCAGGTCGACCTAGCTCCTGCTGCGGCGG |
| *SiRSZ21A* | CATGGAGGCCGAATTCATGGCCCGCTTGTACGTCG | TCCCGTATCGATCTAGCTCCTGCTGCGGCGG |
| **Site Directed Mutagenesis and BiFC assays** | | |
| *SiRSZ21A* | GAAGGCGCCGAGCTAGGAGTCGAAG | CTTCGACTCCTAGCTCGGCGCCTTC |
| *SiRSZ21A* | CCGAAGCAGGGCACGAAGCCGCA | TGCGGCTTCGTGCCCTGCTTCGG |
| *SiRSZ21A* | GAAGCCGCGCGCGCAGTCCC | GGGACTGCGCGCGCGGCTTC |
| *SiGlyoxalase* | GGATCCATGAGGGCTCTCCCGACGA | CTCGAGCTCCAGCTCCTTGGCAAAG |
| *SiUniversal stress protein* | GTCGACATGGCCGGCGCCGGGGCGG | CTCGAGCTTTCCCTTGACGACTGTCAC |
| *SiDomain of unknown function* | GGATCCATGGATCTATCACATTCCC | GTCGACCACCTGATTAATGGAGATGAG |
| *Si14-3-3_f* | GGATCCATGGCATCAGCAGAGCTTTC | CTCGAGCTGCCCCTCGCTCGAGTCGT |
