# Supplementary material for: Unraveling 14-3-3 Proteins in C4 Panicoids with Emphasis on Model Plant Setaria italica Reveals Phosphorylation-Dependent Subcellular Localization of RS Splicing Factor
Source: PLoS One. 2015 Apr 7;10(4):e0123236. doi: 10.1371/journal.pone.0123236 (PMC4388342; doi:10.1371/journal.pone.0123236)
Supplement: S5 Table — (DOC) [file pone.0123236.s009.doc]

**Table S5. Summary of tandemly and segmentally duplicated gene pairs and its evolutionary significance.**

| **SEGMENTALLY DUPLICATED GENES IN MAIZE** | | | | | | | | | | | |
| --- | --- | --- | --- | --- | --- | --- | --- | --- | --- | --- | --- |
| **Gene 1** | **Chromosome** | **Start** | **End** | **Gene 2** | **Chromosome** | **Start** | **End** | **Ka** | **Ks** | **Ka/Ks** | **Time of duplication (MYA)** |
| Zm14-3-3_b | 1 | 196244790 | 196248367 | Zm14-3-3_t | 6 | 157578818 | 157581200 | 0.01 | 0.09 | 0.11 | 6.9 |
| Zm14-3-3_c | 1 | 214899491 | 214901246 | Zm14-3-3_m | 4 | 81702626 | 81706239 | 0.02 | 0.13 | 0.15 | 10.0 |
| Zm14-3-3_h | 2 | 211175628 | 211184411 | Zm14-3-3_y | 8 | 145658003 | 145667670 | 0.01 | 0.11 | 0.09 | 8.5 |
| **Mean** | | | | | | | | **0.01** | **0.11** | **0.12** | **8.5** |
| **SEGMENTALLY DUPLICATED GENES IN FOXTAIL MILLET** | | | | | | | | | | | |
| **Gene 1** | **Chromosome** | **Start** | **End** | **Gene 2** | **Chromosome** | **Start** | **End** | **Ka** | **Ks** | **Ka/Ks** | **Time of duplication (MYA)** |
| Si14-3-3_a | 1 | 28883885 | 28885527 | Si14-3-3_f | 7 | 22066645 | 22070182 | 0.03 | 0.25 | 0.09 | 19.2 |
| Si14-3-3_c | 6 | 27582937 | 27586480 | Si14-3-3_f | 7 | 22066645 | 22070182 | 0.02 | 0.24 | 0.06 | 18.5 |
| **Mean** | | | | | | | | **0.03** | **0.25** | **0.08** | **18.8** |
| **TANDEMLY DUPLICATED GENES IN MAIZE** | | | | | | | | | | | |
| **Gene 1** | **Chromosome** | **Start** | **End** | **Gene 2** | **Chromosome** | **Start** | **End** | **Ka** | **Ks** | **Ka/Ks** | **Time of duplication (MYA)** |
| Zm14-3-3_e | 2 | 32496378 | 32499262 | Zm14-3-3_f | 2 | 41303279 | 41306867 | 0.01 | 0.12 | 0.08 | 9.2 |
| Zm14-3-3_o | 4 | 193608975 | 193612427 | Zm14-3-3_p | 4 | 196903412 | 196904780 | 0.02 | 0.13 | 0.15 | 10.0 |
| Zm14-3-3_r | 6 | 75101749 | 75103690 | Zm14-3-3_s | 6 | 81915950 | 81917882 | 0.01 | 0.1 | 0.10 | 7.7 |
| **Mean** | | | | | | | | **0.01** | **0.12** | **0.11** | **9.0** |
