# Supplementary material for: Unraveling 14-3-3 Proteins in C4 Panicoids with Emphasis on Model Plant Setaria italica Reveals Phosphorylation-Dependent Subcellular Localization of RS Splicing Factor
Source: PLoS One. 2015 Apr 7;10(4):e0123236. doi: 10.1371/journal.pone.0123236 (PMC4388342; doi:10.1371/journal.pone.0123236)
Supplement: S7 Table — (DOC) [file pone.0123236.s011.doc]

**Table S7.** Details of Si14-3-3 orthologs in sorghum, maize, rice and *Brachypodium* and its evolutionary significance.

| ***Setaria italica*** | | | | ***Sorghum bicolor*** | | | | **Ka** | **Ks** | **Ka/Ks** | **Mya** |
| --- | --- | --- | --- | --- | --- | --- | --- | --- | --- | --- | --- |
| **ID** | **Chr** | **Start** | **End** | **Gene ID** | **Chr** | **Start** | **End** |
| Si14-3-3_d | 6 | 31191594 | 31194344 | Sb07g029110.1 | Chr07 | 64102766 | 64103236 | 0.04 | 0.27 | 0.14 | 20.92 |
| Si14-3-3_e | 6 | 34200200 | 34203484 | Sb07g020990.2 | Chr07 | 60708932 | 60708096 | 0.02 | 0.28 | 0.06 | 21.54 |
| Si14-3-3_f | 7 | 22066645 | 22070182 | Sb06g019100.1 | Chr07 | 60708932 | 60708096 | 0.00 | 0.21 | 0.02 | 16.43 |
| Si14-3-3_g | 8 | 28259836 | 28263062 | Sb05g021020.1 | Chr07 | 60708929 | 60708096 | 0.02 | 0.27 | 0.07 | 20.83 |
| Si14-3-3_h | 8 | 33310402 | 33317983 | Sb05g024160.1 | Chr05 | 57057247 | 57056945 | 0.04 | 0.30 | 0.13 | 22.88 |
| **Mean** | | | | | | | | **0.02** | **0.27** | **0.09** | **20.52** |

| ***Setaria italica*** | | | | ***Zea mays*** | | | | **Ka** | **Ks** | **Ka/Ks** | **Mya** |
| --- | --- | --- | --- | --- | --- | --- | --- | --- | --- | --- | --- |
| **ID** | **Chr** | **Start** | **End** | **Gene ID** | **Chr** | **Start** | **End** |
| Si14-3-3_a | 1 | 28883885 | 28885527 | GRMZM2G078641_T01 | 2 | 41306072 | 41305248 | 0.04 | 0.28 | 0.13 | 21.54 |
| Si14-3-3_d | 6 | 31191594 | 31194344 | GRMZM2G140545_T01 | 4 | 1.97E+08 | 1.97E+08 | 0.04 | 0.24 | 0.17 | 18.46 |
| Si14-3-3_e | 6 | 34200200 | 34203484 | GRMZM2G091155_T02 | 4 | 1.97E+08 | 1.97E+08 | 0.02 | 0.29 | 0.06 | 22.31 |
| Si14-3-3_g | 8 | 28259836 | 28263062 | GRMZM2G408768_T01 | 4 | 1.97E+08 | 1.97E+08 | 0.02 | 0.27 | 0.08 | 20.77 |
| Si14-3-3_h | 8 | 33310402 | 33317983 | GRMZM2G145213_T01 | 4 | 5966331 | 5966029 | 0.04 | 0.26 | 0.15 | 19.72 |
| **Mean** | | | | | | | | **0.03** | **0.27** | **0.12** | **20.56** |

| ***Setaria italica*** | | | | ***Oryza sativa*** | | | | **Ka** | **Ks** | **Ka/Ks** | **Mya** |
| --- | --- | --- | --- | --- | --- | --- | --- | --- | --- | --- | --- |
| **ID** | **Chr** | **Start** | **End** | **Gene ID** | **Chr** | **Start** | **End** |
| Si14-3-3_a | 1 | 28883885 | 28885527 | LOC_Os02g36974.4 | Chr4 | 23104855 | 23105265 | 0.03 | 0.46 | 0.06 | 35.27 |
| Si14-3-3_d | 6 | 31191594 | 31194344 | LOC_Os08g37490.1 | Chr8 | 23750428 | 23750874 | 0.03 | 0.45 | 0.07 | 34.62 |
| Si14-3-3_e | 6 | 34200200 | 34203484 | LOC_Os08g33370.2 | Chr4 | 23104873 | 23105265 | 0.03 | 0.57 | 0.06 | 43.85 |
| Si14-3-3_f | 7 | 22066645 | 22070182 | LOC_Os04g38870.5 | Chr4 | 23104855 | 23105265 | 0.01 | 0.44 | 0.03 | 33.89 |
| Si14-3-3_g | 8 | 28259836 | 28263062 | LOC_Os04g38870.5 | Chr4 | 23104855 | 23105265 | 0.26 | 0.48 | 0.54 | 36.92 |
| **Mean** | | | | | | | | **0.07** | **0.48** | **0.15** | **36.91** |

| ***Setaria italica*** | | | | ***Brachypodium distachyon*** | | | | **Ka** | **Ks** | **Ka/Ks** | **Mya** |
| --- | --- | --- | --- | --- | --- | --- | --- | --- | --- | --- | --- |
| **ID** | **Chr** | **Start** | **End** | **Gene ID** | **Chr** | **Start** | **End** |
| Si14-3-3_a | 1 | 28883885 | 28885527 | Bradi5g12510.2 | Bd3 | 48692570 | 48692983 | 0.03 | 0.83 | 0.03 | 63.69 |
| Si14-3-3_b | 5 | 25767301 | 25771639 | Bradi3g36480.2 | Bd3 | 38831429 | 38830875 | 0.04 | 0.80 | 0.04 | 61.86 |
| Si14-3-3_d | 6 | 31191594 | 31194344 | Bradi3g38640.1 | Bd3 | 41142646 | 41142176 | 0.05 | 0.78 | 0.06 | 60.00 |
| Si14-3-3_g | 8 | 28259836 | 28263062 | Bradi4g16640.1 | Bd4 | 17459509 | 17459084 | 0.04 | 0.82 | 0.05 | 63.08 |
| Si14-3-3_h | 8 | 33310402 | 33317983 | Bradi4g13970.2 | Bd4 | 14438129 | 14438464 | 0.07 | 0.81 | 0.09 | 62.62 |
| **Mean** | | | | | | | | **0.04** | **0.81** | **0.06** | **62.25** |
